# Genetics of Local Adaptation in the Laboratory: Flowering Time Quantitative Trait Loci under Geographic and Seasonal Conditions in *Arabidopsis*

**DOI:** 10.1371/journal.pone.0000105

**Published:** 2006-12-27

**Authors:** Yan Li, Peter Roycewicz, Evadne Smith, Justin O. Borevitz

**Affiliations:** 1 Department of Ecology and Evolution, University of Chicago, Chicago, Illinois, United States of America; 2 Committee on Genetics, University of Chicago, Chicago, Illinois, United States of America; University of California, Davis, United States of America

## Abstract

Flowering time in *Arabidopsis thaliana* is controlled by a large number of genes and various environmental factors, such as light and temperature. The objective of this study was to identify flowering time quantitative trait loci (QTL) under growth conditions simulating seasonal conditions from native geographic locations. Our growth chambers were set to simulate the spring conditions in Spain and Sweden, with appropriate changes in light color, intensity and day length, as well as temperature and relative humidity. Thus the Sweden-like spring conditions changed more dramatically compared to Spain-like spring conditions across the duration of our experiment. We have used these conditions to map QTL responsible for flowering time in the Kas-1/Col-*gl1* recombinant inbred lines (RILs) across two replicate blocks. A linkage map from 96 RILs was established using 119 markers including 64 new SNPs markers. One major QTL, mapping to the *FRIGIDA* (*FRI*) locus, was detected on the top of chromosome 4 that showed significant gene×seasonal environment interactions. Three other minor QTL also were detected. One QTL mapping near *FLOWERING LOCUS M* (*FLM*) showed an epistatic interaction with the QTL at *FRI*. These QTL×environment and QTL×QTL interactions suggest that subtle ecologically relevant changes in light, temperature, and relative humidity are differentially felt by alleles controlling flowering time and may be responsible for adaptation to regional environments.

## Introduction

Flowering time is a critical step in the life of annual species such as *Arabidopsis thaliana*. The proper timing of reproduction to coincide with suitable environments is important for its survival and interactions with other ecological factors. The major environmental cues that affect flowering time are light and temperature, which change daily through seasons and vary with geographic locations. The diversity of flowering time and responses to environmental variables in wild accessions of *A. thaliana* [1]–[3] suggest that *A. thaliana* has precise and diversified mechanisms controlling flowering time across geographic locations and seasons.

*A. thaliana* provides a great opportunity to determine the genetic basis of flowering time and response to environments. Many genes controlling flowering time have been identified (see the flowering web http://www.salk.edu/LABS/pbio-w/flower_web.html and reviews by [4]–[7]), and a genetic network has been outlined which includes four main pathways: photoperiod, vernalization, autonomous, and gibberellin [8]. Longer days act via the photoperiod pathway and an extended period of winter-like temperature act via the vernalization pathway to accelerate flowering time by releasing repression caused by the floral inhibitor *Flowering Locus C (FLC)*. Functional alleles of another gene, *FRIGIDA (FRI)*, promote the accumulation of *FLC* mRNA, which delays flowering. The autonomous pathway also promotes flowering by negatively regulating *FLC*. Ambient growth temperature may affect flowering time through an autonomous pathway [9]. In addition to *FLC*, another MADS (MCM1/AGAMOUS/DEFICIENS/SRF1)-box gene, *FLOWERING LOCUS M (FLM)*, similar in amino-acid sequence to *FLC*, also acts as an inhibitor of flowering in the Ws accession [10]. The Col allele at *FLM* delays flowering compared to the null *flm*, including the natural null allele in the Nd accession [11].

Natural genetic variation in *A. thaliana* for flowering response under different environmental conditions has been observed including photoperiod, vernalization and ambient temperature [1]–[3], [12], [13]. A latitudinal cline in flowering time was observed in the European accessions of *A. thaliana* when grown over winter in a common garden in Rhode Island. This cline however was only seen in accessions that lacked common *FRI* deletion polymorphisms [3]. This may be due to a latitudinal cline in vernalization sensitivity: the southern accessions were more sensitive to vernalization than the northern accessions [2]. Since environmental factors such as light and temperature are correlated with latitude, the latitudinal clines for flowering time and vernalization sensitivity are suggestive of natural selection acting on flowering time. Direct tests are difficult as field studies are influenced by many random factors. It may be difficult to see modest genetic effects of evolutionary significance and when identified may correspond to the particulars of a given year rather than the standard local conditions.

Generally, flowering time is accelerated by longer days, higher temperature [14], and vernalization, conditions associated with the more favorable conditions of spring and summer. How plants integrate signals from these environmental cues to decide when to flower is essential to survival. In natural conditions, a major environmental cue is the continuous seasonal changes of light, including the sunrise and sunset times perceived as changes in light quality and intensity. Such dynamic changes are distinct from traditional studies of fixed photoperiod, light quality and intensity. In nature, temperature also cycles during the day and changes predictably during the season, in coordination with light. These seasonal changes in light and temperature are a unique fingerprint of a particular geographic location. Standard lab experiments of long day vs. short day, with and without vernalization at constant temperature do not capture the changing seasonal patterns unique to local conditions. Thus to identify the loci involved in local adaptation to predictable seasonal environmental cues of day length, temperature, light color, and humidity, experiments should be performed under controlled settings simulating these multivariate conditions.

Our experiments aim to recreate seasonal conditions to study the flowering time QTL and their interactions with geographic locations within the spring season. This general approach of simulating natural conditions in the lab provides a compromise between simple environmental contrasts in the lab and the unpredictable complexity of field studies. However, the demanding dual strategy of lab and field studies is also warranted [15], [16]. We used Kas-1/Col-*gl1* recombinant inbred lines (RILs) (Somerville lines) in this study as a test case before moving to local populations. The parents Kas-1 and Col-*gl1* differ in flowering time [17]. The results confirmed the quantitative nature of genetic variation in flowering time and the major role of polymorphisms in the *FRI* gene in flowering time variation. QTL×environment interactions suggested that seasonal changes affect flowering time differentially via alleles known to control flowering time. We propose that such interactions may be responsible for adaptation to regional environments. Additionally, epistasis between the loci near *FLOWERING LOCUS M* (*FLM*) and *FRI* suggest a novel interaction between loci known to control natural variation of flowering time in *A. thaliana*.

## Materials and Methods

### Growth Chambers

**Figure 1:**
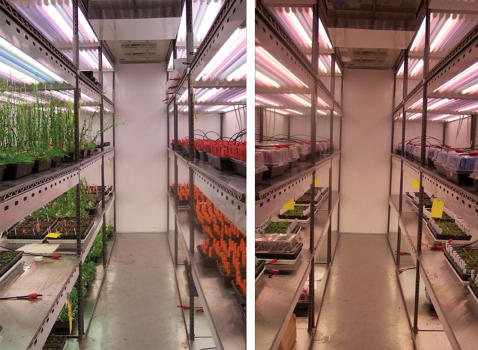
Side by side walk in Growth Chambers running Sweden (left) and Spain (right) simulated seasonal conditions. Days until flowering was recorded for 1123 plants in total seen growing in two blocks on the right and left side of each chamber.

Experiments were conducted within two walk-in growth chambers (AR-916, Percival Scientific, Inc. Perry, IA) that were programmed to simulative ideal seasonal conditions in Madrid, Spain and Stockholm, Sweden, respectively (Fig. 1). Sunrise and sunset, light spectrum, temperature and relative humidity were programmed to cycle throughout the day and the season according to 30 year seasonal averages (modeled as described in the supplemental data http://naturalvariation.org/KasCol). The experiment began on May 1^st^ settings in both Spain and Sweden. In brief, sunrise and sunset changed every day throughout the season, light intensity and spectral mixture changed every six minutes throughout the day as did temperature and relative humidity. Sunrise and sunset times were generated from mathematical models as a function of latitude, longitude, time zone, and day of year (http://www.sci.fi/∼benefon/sun.php3). The light quality spectrum and intensity were recreated using far-red (700–750 nm), red (630–700 nm), cool-white (520–600 nm), and blue (400–500 nm) fluorescent bulbs on electronically dimmable ballasts based on the solar spectrum model. The ratio of blue light to red light varied from 0.2 at sunrise to 1.1 at noon and the ratio of red light to far-red light varied from 0.95 at sunrise to 0.65 at noon. Daily temperature variation was modeled based on a general empirical profile fitted over several days. Temperature increased at sunrise, reached maximum shortly after noon and decreased again until sunrise. The amplitude of the curve spans the daily minimum and maximum temperature for the location of interest. Data on minimum and maximum daily temperatures were obtained from Weather.com. The relative humidity changed with temperature maintaining constant total humidity.

### Plant Materials

A set of 128 F_6_ Kas-1/Col-*gl1* recombinant inbred lines (RILs) and the two parental lines (Somerville lines) were obtained from the Arabidopsis Biological Resource Center (Columbus, OH). Fourteen lines were reordered from the Arabidopsis Biological Resource Center (ABRC) due to an inconsistency between previous studies [17], [18] and 96 lines were included in the final data set.

### Experimental design and flowering time measurements

The two walk-in growth chambers mimiced two geographic locations, Sweden and Spain. There were two blocks in each location labeled Sweden 1, Sweden 2, Spain 1, and Spain 2. Seeds were stratified for 24 hours at 4°C. Initially one to two seeds were planted in each pot of the 4×9-well flat and thinned to a single seedling after germination. Within each block, 12 lines were planted per 4×9-cell flat, with 3 pots for each line. Lines were arranged randomly across flats. In total, 12 flats contained 142 lines (128 plus the 14 duplicate lines). This set of 12 flats was replicated an additional 3 times so that four sets of 12 flats were placed in the four blocks. Flats were rotated within the block at a rate of approximately 2 flats per week. Individual pots in each flat were also occasionally rotated within the flat. Plants were watered daily by hand and additional water was provided using an automatic watering system. Each single plant in a pot was bar-coded with a unique identifier which allowed individual tracking of flowering time measured as days to flowering (DTF) i.e. the number of days from sowing until the appearance of the first floral buds monitored almost daily.

### DNA extraction and SNP genotyping

Genomic DNA was extracted using MagAttract 96 DNA Plant Kit (Qiagen Inc., Valencia, CA) and KingFisher 96 (Thermo Electron Corporation, Waltham, MA). One plant from each line of the RIL set including the parents and the duplicate lines were genotyped at 65 single nucleotide polymorphism markers by Sequenom (San Diego, CA). Markers were a subset of those described at http://naturalvariation.org/geno.html.

### Preparation of data files

After SNP genotyping, one marker was dropped from further analysis because of severe segregation distortion likely due to duplicate amplification. After cluster analysis (hclust in R, complete linkage method) of the genotypic data generated from 64 SNP markers, lines with genotypes inconsistent with previous reports [17], [18] were removed and those with identical genotypes were merged. 18 lines had inconsistent genotypes or were likely to be mislabeled and were excluded from the further analysis: CS84873, C84875, CS84902, CS84904, CS84907, CS84910, CS84912, CS84914, CS84924, CS84925, CS84930, CS84947, CS84970, CS84974, CS84981, CS84991, CS84992, and CS84993. Two lines had identical genotypes (CS84943 = CS84945). The phenotypes for identical lines (including the five duplicate lines and the two identical genotype lines) were merged together. Three lines with less than six measurements of the phenotypic data were also excluded from further analysis. Finally 96 RILs in total were used in the genetic linkage map and QTL mapping. Marker genotypes and raw phenotypic data are available as supporting information at http://naturalvariation.org/KasCol.

### Genetic map

The genotypic data for the 96 RILs from the 64 SNP markers and the previous 55 markers [18] was analyzed together using GMendel 3.0 [19]. Most of the marker orders were consistent with the physical map (AGI sequence map) of the Col sequence (see supporting information). Map distances were obtained from GMendel 3.0 using the Kosambi map function. The final map was rendered using MapChart 2.1 [20].

### QTL analyses

QTL analyses were performed in bQTL, R package (http://hacuna.ucsd.edu/bqtl). Interval mapping and analysis of posterior probabilities using Bayes model averaging over different, multigene QTL models were carried out separately in the four blocks (Table 1). We present the results of the multigene linear Bayes models with 2-cM scanning resolution. The false discovery rate (FDR) posterior odds threshold was obtained by sorting the results of 400 genotype phenotype permutations scans [21]. Four QTL were detected at 1% FDR, SNP21607030 (Chr1), SNP21607175 (Chr3), MSAT4.39 (Chr4), and SNP44607955 (Chr4). These were included as main effect background markers in subsequent complete pairwise epistasis scans performed in each of four blocks. Significant epistasis was identified in Spain 2 at a *P*<0.05 genome-wise threshold set by 500 permutations. In the final analysis, QTL, environment, QTL×QTL, and QTL×environment interactions were included as fixed effects with block and flat as nested random effects in a single linear mixed-effects model testing each plant separately. RIL was not treated as a random factor in this model. The R scripts, raw data, and other supporting materials are available at http://naturalvariation.org/KasCol.

**Table 1:**
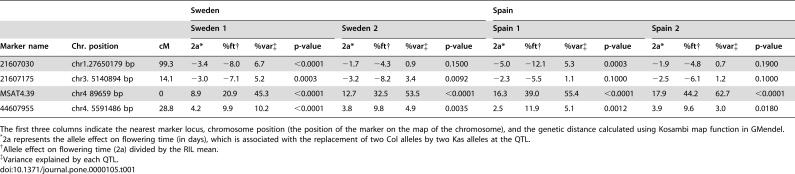
QTL effects on flowering time in four environments (blocks).

			Sweden								Spain							
			Sweden 1				Sweden 2				Spain 1				Spain 2			
Marker name	Chr. position	cM	2a*	%ft†	%var‡	p-value	2a*	%ft†	%var‡	p-value	2a*	%ft†	%var‡	p-value	2a*	%ft†	%var‡	p-value
21607030	chr1.27650179 bp	99.3	−3.4	−8.0	6.7	<0.0001	−1.7	−4.3	0.9	0.1500	−5.0	−12.1	5.3	0.0003	−1.9	−4.8	0.7	0.1900
21607175	chr3. 5140894 bp	14.1	−3.0	−7.1	5.2	0.0003	−3.2	−8.2	3.4	0.0092	−2.3	−5.5	1.1	0.1000	−2.5	−6.1	1.2	0.1000
MSAT4.39	chr4 89659 bp	0	8.9	20.9	45.3	<0.0001	12.7	32.5	53.5	<0.0001	16.3	39.0	55.4	<0.0001	17.9	44.2	62.7	<0.0001
44607955	chr4. 5591486 bp	28.8	4.2	9.9	10.2	<0.0001	3.8	9.8	4.9	0.0035	2.5	11.9	5.1	0.0012	3.9	9.6	3.0	0.0180

The first three columns indicate the nearest marker locus, chromosome position (the position of the marker on the map of the chromosome), and the genetic distance calculated using Kosambi map function in GMendel.

* 2a represents the allele effect on flowering time (in days), which is associated with the replacement of two Col alleles by two Kas alleles at the QTL.

† Allele effect on flowering time (2a) divided by the RIL mean.

‡ Variance explained by each QTL.

## Results

### Genetic map in Kas-1/Col-*gl1* RILs

**Figure 2:**
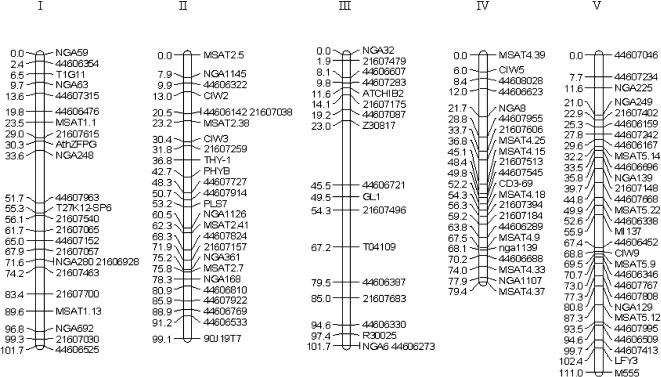
Genetic map of the Kas-1×Col-*gl1* RIL population. Distances are in centimorgans using the Kosambi map function. Data for the 55 markers from Wolyn et al. (2004) were merged with 64 SNP markers.

A linkage map (Fig. 2) was established using 119 markers including 64 new SNP loci and 55 previously genotyped markers [18] among 96 Kas-1/Col-*gl1* RILs. The total genetic distance covered by these 119 markers was 492.9 cM with the largest gap of 22.5 cM on chromosome 3. Forty-six (39%) markers showed segregation distortion (*P*<0.05), especially on three regions: the top of chromosomes I and IV, and the middle of chromosome III, where more lines with Col-*gl1* alleles than Kas-1 alleles in these three regions. Significant segregation distortion of some regions in the Kas-1/Col-*gl1* RILs seen here, were also detected in previous studies [17], [18]. Segregation distortions of markers have also been seen in other *A. thaliana* RIL populations [13], [22], [23]. This distortion could be due to inadvertent selection of the lines in this mapping population, biases in genotyping error, and/or due to loci containing underlying sterility or incompatibility factors [24]–[27].

### Flowering-time variation in Kas-1/Col-*gl1* RILs

**Figure 3:**
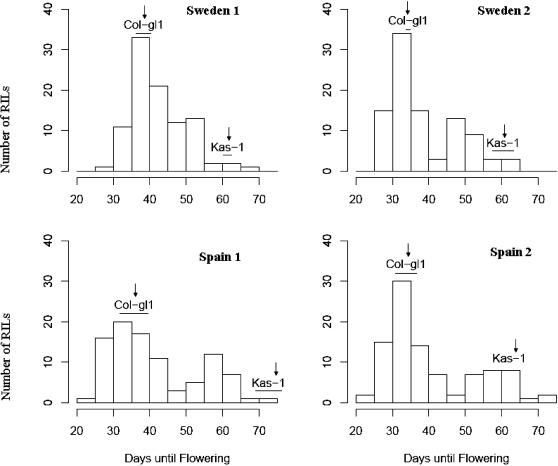
Distribution of flowering time among 96 Kas-1×Col-*gl1* RILs. Flowering time was measured as days until flowering in Sweden (upper) and Spain (lower) across two replicated blocks. The means and standard deviations of parents (Col-*gl1* and Kas-1) are shown by arrows and lines, respectively.

The histograms of the RIL flowering-time reveal continuous, quantitative genetic variation with greater variation in the Spain environment (Fig. 3). The parental line Kas-1 flowered later than Col-*gl1* (63.6±5.5 vs. 35.5±2.9 in days) across the four experimental blocks. An analysis of variance showed that the flowering-time was significantly different among the 96 Kas-1/Col-*gl1* RILs (*P*<0.01) but the main effect of two “geographic locations”, Sweden and Spain (*P>*0.05) was not. Significant interactions of RILs×geographic locations and RILs×blocks were detected (*P*<0.01). Since the block effect was significant, QTL were separately mapped under the four block environments in the following analyses.

### Identification of flowering-time QTL, gene×environment interactions, and epistasis

**Figure 4:**
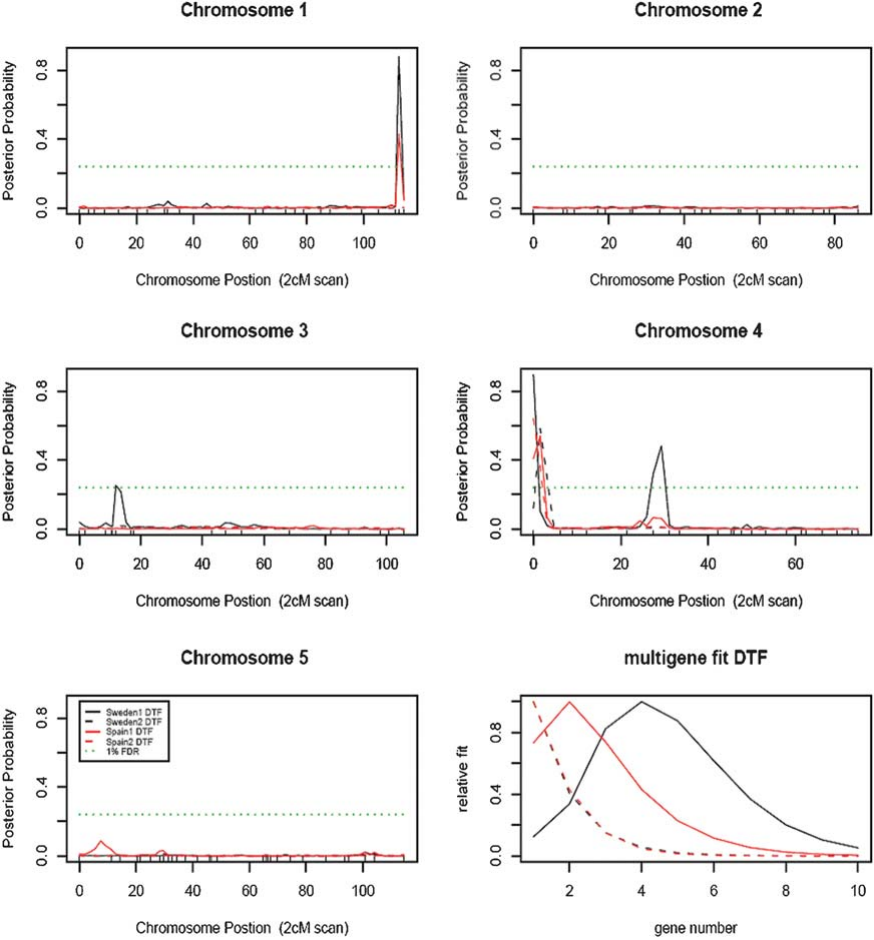
QTL map of days to flower (DTF) in Sweden (black line) and Spain (red line). Analysis of posterior probabilities was based on Bayes model and the interval at each point represented 2-cM scanning resolution. Solid lines show block 1 and dashed lines show block 2 each modeled separately. The dotted green horizontal line corresponds to 1% false discovery rate (FDR) set by 400 permutations. The multigene model averaged Bayesian QTL model fits 1 to 10 genes models. The relative weight of each model is shown (see R/bQTL package and supplementary data for a more detailed explanation).

BQTL mapping was carried out independently in four blocks since blocks within Sweden and Spain environments had significant effects. Four QTL were detected using Linear Bayes analyses at 1% FDR (Fig. 4), and their effects were estimated separately in the four individual blocks (Table 1) or together in the full model (Table 2, analysis details described in the methods). Kas alleles delayed flowering at two QTL (chr4. 89659 bp and chr4. 5591486 bp) while promoted flowering at the other two QTL (chr1. 27650179 bp and chr3. 5140894 bp) compared to Col alleles. We next tested all possible marker and 1 cM marker interval pairs for interactions with experiment-wise threshold of *P* = 0.05 by 500 permutations [28]. A significant epistasis was identified between a marker at the QTL on chromosome 1, SNP 21607030 which is 1.3 Mbp above *FLM*, and a marker at the first chromosome 4 QTL, MSAT 4.39 which is 0.18 Mbp above the *FRI* locus.

**Table 2:**
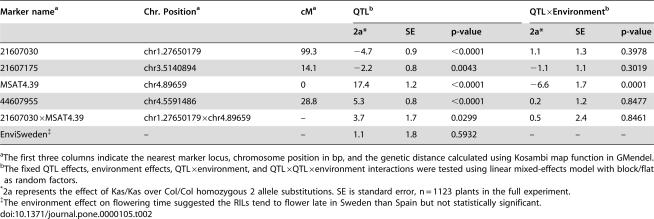
The effects of QTL and QTL×Environment on flowering time in the final model.

Marker name^a^	Chr. Position^a^	cM^a^	QTL^b^			QTL×Environment^b^		
			2a*	SE	p-value	2a*	SE	p-value
21607030	chr1.27650179	99.3	−4.7	0.9	<0.0001	1.1	1.3	0.3978
21607175	chr3.5140894	14.1	−2.2	0.8	0.0043	−1.1	1.1	0.3019
MSAT4.39	chr4.89659	0	17.4	1.2	<0.0001	−6.6	1.7	0.0001
44607955	chr4.5591486	28.8	5.3	0.8	<0.0001	0.2	1.2	0.8477
21607030×MSAT4.39	chr1.27650179×chr4.89659	–	3.7	1.7	0.0299	0.5	2.4	0.8461
EnviSweden‡	–	–	1.1	1.8	0.5932	–	–	–

a The first three columns indicate the nearest marker locus, chromosome position in bp, and the genetic distance calculated using Kosambi map function in GMendel.

b The fixed QTL effects, environment effects, QTL×environment, and QTL×QTL×environment interactions were tested using linear mixed-effects model with block/flat as random factors.

* 2a represents the effect of Kas/Kas over Col/Col homozygous 2 allele substitutions. SE is standard error, n = 1123 plants in the full experiment.

‡ The environment effect on flowering time suggested the RILs tend to flower late in Sweden than Spain but not statistically significant.

**Figure 5:**
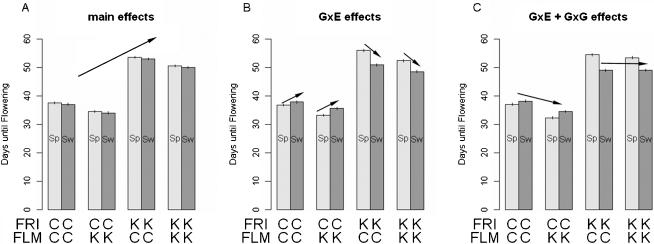
Main effects, Genotype×Environment, and Epistasis. Effects described below are illustrated by the arrows in each panel A) Modeling the main effect of *FRI* shows the large floral repressing effect of the Kas *FRI* allele. B) Modeling the environmental interaction shows that Kas *FRI* has less of an effect in the cooler Sweden conditions. C) Including the interaction term for *FLM*×*FRI* reveals that in the Col *fri* background Kas *FLM* promote flowering while in the Kas *FRI* background Kas *FLM* has little effect. Error bars show 95% confidence intervals estimated from the standard residual of each model across 1123 plants in the experiment. Abbreviations: Sp (Spain), Sw (Sweden), C (Col), K (Kas).

The QTL and environment main effects, QTL×QTL interaction, and all QTL×environment interactions were tested in the final linear mixed-effects model with block/flat as random factors (Table 2). The four QTL were still significant in the joint analysis (*P*<0.05). The major QTL near *FRI* on chromosome 4 showed a strong QTL×environment and minor QTL×QTL interaction (Table 2). Col is known to carry a deletion at *FRI* [13]. In Figure 5, three models are presented showing that *FRI*'s effect can be detected as a main effect (fig 5A) but also responds to both environment (Fig. 5B) and genotype (Fig. 5C). The likely functional Kas allele at this QTL delayed flowering-time by 17.4 days in Spain as compared to the Col loss of function allele (Table 2, Fig 5A). In the colder Sweden-like environment the effect of the functional Kas allele was reduced by 6.6-days. Thus the magnitude of *FRI*'s effect is environment-dependant, and Kas *FRI*'s effect is reduced in the colder Sweden conditions (Fig. 5B). Additionally, the presence of an effect of *FLM* on flowering time was dependant on *FRI* genotype (Fig. 5C). Kas *FLM* promotes flowering in a Col- *fri* null background, but has little effect when in a functional Kas-*FRI* background.

## Discussion

In this study, simulated seasonal environments resembling Sweden and Spain with variable diurnal light, temperature, and relative humidity were set up in two walk-in growth chambers. Under these conditions, four QTL responsible for the difference in spring flowering-time between Kas-1 and Col-*gl1* were mapped in 96 Kas-1/Col-*gl1* RILs using 119 molecular markers. The Kas allele of a large effect QTL at the *FRIGIDA* (*FRI*) locus, delayed flowering time as did another minor QTL, while at two other minor QTL Col alleles delayed flowering time. A significant QTL×environment interaction was detected at *FRI*. The effect was dampened in the cooler Sweden-like environment implying that subtle changes in light quality, rate of change of day length, and temperature are differentially integrated at the genetic level and thus may be responsible for adaptation to regional abiotic cues. The Kas-1 *FRI* allele does not carry common deletions while Col-*gl1 FRI* carries a loss of function deletion polymorphism [13]. It is very likely that the *FRI* indel polymorphism is the molecular basis of the first chromosome 4 QTL (Figure 4).

The significant epistasis between markers SNP 21607030 and MSAT 4.39 suggests a potential interaction between the alleles of *FLM* and *FRI*, which has not been reported before. *FLM* is a MADS-domain gene on chromosome 1 and delays flowering time similar to it close homologue the *FLC* MADS-domain gene [10]. Our earlier study [11] showed that Col *FLM* delays flowering compared to null *flm* alleles, such as found in the Nd ecotype. In the present study, the Kas *FLM* allele lacks floral repressor function compared with Col *FLM*, similar to the Nd null allele. This allelic variation however is only seen in the Col- *fri* null allele background which differs from the well known positive effect of *FRI* on *FLC*. *FLM* is a candidate gene for the chromosome 1 QTL; however, on the Col reference sequence, the NGA692 marker is slightly closer to *FLM* (∼120 kb below) compared to SNP 21607030 marker, but is less tightly linked with the chromosome 1 QTL. The epistasis between markers linked to the *FLM* and *FRI* loci is suggestive and warrants further study. Creating near isogenic lines that differ for the Kas and Col alleles at *FLM* in functional and non-functional *FRI* backgrounds would allow us to confirm the predicted genetic basis of the epistasis identified in this study. If confirmed, the epistatic relation described here between *FRI* and *FLM* reveals a novel regulatory evolution that is similar to the *FRI-FLC* pair [29], [30].

The QTL and QTL×environment interaction detected in this study suggest that natural-like environments can reveal potentially ecologically important variation in relevant traits such as flowering time. In addition to contrasting geographic locations at ranges of latitudes as we have done here, the approach can be applied to other contrasts. For example seasons (e.g. spring and fall) can be compared thus allowing experimentation addressing the basis of the life history strategies of winter and summer annual growth habits. We believe that abiotic seasonal and geographic cues are the major predictable local environmental signals to which *A. thaliana* must adapt in order to succeed. Thus, quantitative trait loci identified under such conditions are more likely to be ecologically relevant and reflect the genetic basis of differences in life history between locally adapted populations.
